# Atypical Time Course of Object Recognition in Autism Spectrum Disorder

**DOI:** 10.1038/srep35494

**Published:** 2016-10-18

**Authors:** Laurent Caplette, Bruno Wicker, Frédéric Gosselin

**Affiliations:** 1 Département de psychologie, Université de Montréal, Montreal, Canada; 2 Laboratoire de Neurosciences Cognitives, CNRS UMR 7291, Aix-Marseille Université, Fédération 3C, Marseille, France.

## Abstract

In neurotypical observers, it is widely believed that the visual system samples the world in a coarse-to-fine fashion. Past studies on Autism Spectrum Disorder (ASD) have identified atypical responses to fine visual information but did not investigate the time course of the sampling of information at different levels of granularity (i.e. Spatial Frequencies, SF). Here, we examined this question during an object recognition task in ASD and neurotypical observers using a novel experimental paradigm. Our results confirm and characterize with unprecedented precision a coarse-to-fine sampling of SF information in neurotypical observers. In ASD observers, we discovered a different pattern of SF sampling across time: in the first 80 ms, high SFs lead ASD observers to a higher accuracy than neurotypical observers, and these SFs are sampled differently across time in the two subject groups. Our results might be related to the absence of a mandatory precedence of global information, and to top-down processing abnormalities in ASD.

Autism spectrum disorder (ASD) is a neurodevelopmental disorder whose most prominent symptoms are deficits in social interaction and communication, restricted interests and repetitive behaviors. However, ASD is also characterized by sensory and perceptual peculiarities, as research has increasingly demonstrated in recent years^1^. In particular, several studies have reported that ASD subjects are biased toward visual local information compared to global information^2^,^3^,^4^. According to some authors, this is the result of a deficit in the extraction of global aspects of images, which is argued to be a defining feature of autism^5^. Other authors propose that there is no such deficit and that ASD subjects are only better at accessing local information^6^. A recent meta-analysis of these few studies found no strict deficit in global processing or enhanced local processing, but rather only a delay in the processing of global information, suggesting that it is the temporal aspect of this process that is different in ASD^7^. Atypical spatial frequency (SF) processing is also observed in ASD. High SFs are related to local information as they are both particularly well suited to represent small objects in a scene; and low SFs are related to global information as they are both particularly well suited to represent large objects in a scene (they are not equivalent, however, because global information can be retrieved from high SFs). Unlike neurotypicals, individuals with ASD exhibit a greater P1 amplitude in response to high SFs than to low SFs^8^, and a reduced difference between neural responses to high and intermediate SFs^9^,^10^. It was also shown that individuals with ASD use high SFs more than low SFs to recognize faces, while neurotypicals do the opposite^2^. Other studies reported that the contrast sensitivity function (CSF; the low-level visual sensitivity to sinusoidal gratings at different SFs) of ASD subjects peaks at higher SFs than that of neurotypicals^11^,^12^, albeit this remains controversial^13^,^14^.

Importantly, SFs are not sampled all at once during recognition in neurotypicals: they are extracted in a coarse-to-fine manner^15^,^16^. Rapidly extracted low SFs may furthermore guide, in a top-down manner, the subsequent extraction of higher SFs^17^,^18^,^19^. Interestingly, there is evidence that individuals with ASD rely less on top-down mechanisms during perception, when compared to neurotypical individuals, and that this might be crucial in explaining the phenotype of autism^20^,^21^,^22^,^23^. If individuals with ASD do not rely on top-down mechanisms, they might not need to sample SFs in a coarse-to-fine fashion.

To date, however, the time course of SF sampling has never been investigated in ASD. Moreover, most studies demonstrating a coarse-to-fine sampling of SF information in neurotypicals compared a single low SF condition to a single high SF condition, which is unhelpful to uncover the *exact* SFs at play in this process. Here, we used a novel technique based on the Bubbles approach^24^ to map with unprecedented precision the use of SFs across time in both neurotypical and ASD subjects. On each trial, subjects had to recognize an object from a brief video sampling random SFs on random frames; we then reverse correlated the revealed SFs with response accuracy. This technique allows us to reveal the difference between the groups for each SF in each time frame, and thus to settle major issues such as whether ASD subjects exhibit a deficit in sampling low SFs, an enhanced sampling of high SFs, or a difference in the time course of SF sampling.

## Results

There were no difference between groups in average response time (ASD: 814 ms; Controls: 752 ms; *t*(58) = 0.77, *p* > 0.25) or in the average quantity of information (the number of bubbles; see Methods) needed to recognize objects with 75% accuracy (ASD: 42.29; Controls: 40.06; *t*(58) = 0.39, *p* > 0.25).

**Figure 1:**
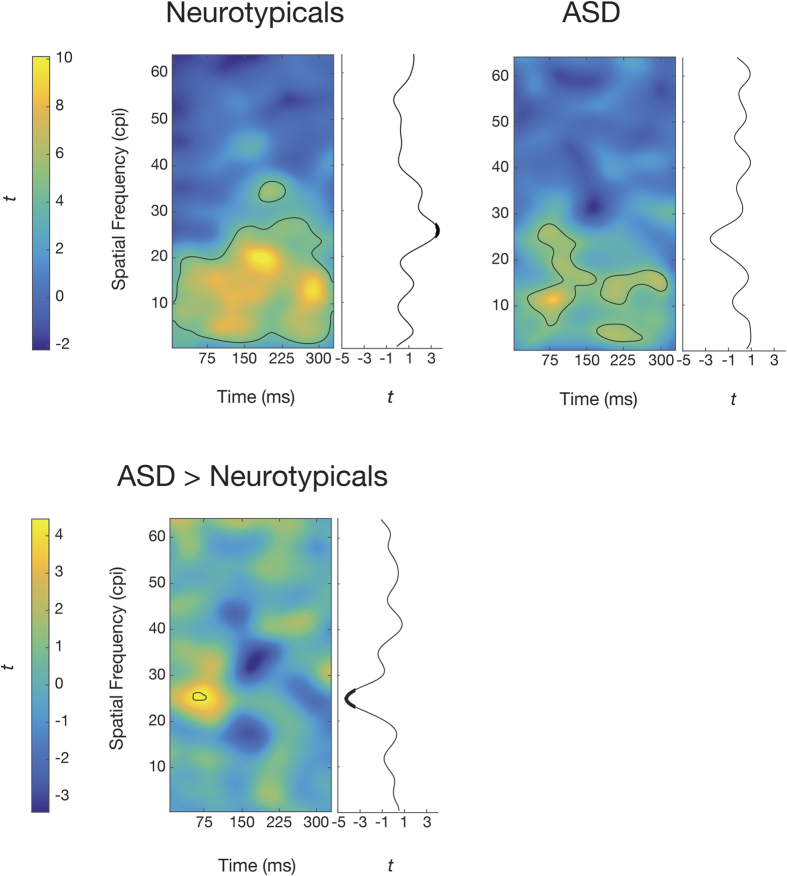
Use of SFs across time in each group and differences between the groups. Upper panel: One-sample *t* maps illustrating how each SF in each time frame correlates with accurate object recognition, and one-sample *t* vectors indicating the slope of use of each SF across time, for the neurotypical and ASD groups. Lower panel: Two-sample *t* map illustrating the between-group differences in the use of each SF in each time frame, and two-sample *t* vector indicating the between-group differences in the slope of use of each SF across time. Pixels enclosed by black lines and bold portions of the vectors are significant (*p* < 0.05, FWER-corrected). Note that since the width of all images subtended 6 degrees of visual angle, cycles per image can be converted in cycles per degree by dividing by 6. Note also that only statistics up to 64 cycles per image (cpi) are shown; there are no statistically significant results in the portions not shown. Note finally that color axes are different in the panels.

To uncover which spatial frequencies in which time frames led to accurate object recognition, we performed multiple least-square linear regressions between accuracies and the SF x time sampling matrices (which indicate if each SF in each time frame is sampled or not) of the corresponding trials, for each subject. Then, for each SF on each time frame, we compared the regression coefficients across subjects and computed one-sample and two-sample *t* statistics; this resulted in within-group and between-group *t* maps indicating respectively how the presentation of specific SFs on specific time frames correlates with accuracy for each group and how these correlations differ between the groups (see 2D plots on Fig. 1).

In neurotypical subjects, SFs between about 1 and 20 cycles per image (cpi) throughout stimulus presentation and SFs up to 36 cpi in the second half of the video (*p* < 0.05, corrected for Family-Wise Error Rate (FWER); peak Cohen’s *d* = 1.60) led to accurate recognition. In ASD subjects, three blobs reached statistical significance (*p* < 0.05, FWER-corrected; peak *d* = 1.90): the largest (291 pixels) peaked at 11 cpi and 80 ms, the second largest (132 pixels) at 15.5 cpi and 296 ms, and the smallest (63 pixels) at 3.5 cpi and 213 ms. In the group contrast, SFs peaking at 25.5 cpi and 71 ms led to more accurate recognition in ASD participants than in neurotypical subjects (*p* < 0.05, FWER-corrected; peak *d* = 1.16; Fig. 1).

To investigate the time course of sampling of each SF and uncover if it was used increasingly or decreasingly across time, we fitted a line across time for each SF in each subject map. We then took the slopes of these lines across subjects and computed one-sample *t* statistics for each SF. This resulted in within-group *t* vectors indicating the slope of use (increasing or decreasing) of each SF across time for each group (Fig. 1, upper panel, 1D plots). To test whether the time course of sampling was different between the groups, we then computed two-sample *t* statistics for each SF. This resulted in a between-group *t* vector indicating how the slopes of use differed between the groups (Fig. 1, lower panel, 1D plots).

Results show that while ASD subjects seem to use each SF in an approximately constant manner across time, neurotypical subjects use SFs around 25 cpi increasingly across time (*p* < 0.05, FWER-corrected; peak *d* = 0.57). These same SFs are used more in an increasing manner across time in neurotypical subjects than in ASD subjects (*p *< 0.05, FWER-corrected; peak *d *= 1.08; Fig. 1). This confirms an interaction between group and time course of sampling of SFs around 25 cpi and shows that this time course differed between the groups for these SFs.

## Discussion

In this study, we investigated with unprecedented precision how neurotypical and ASD subjects sample SFs across time. We first observed that neurotypicals use lower SFs (up to about 20 cpi) throughout all stimulus presentation, while they use higher SFs increasingly with the passage of time. This confirms previous findings of a coarse-to-fine sampling, and nuances them by showing that low SFs continue to be used in the latest time frames. This is the first piece of evidence for a hypothesis originally formulated by Ullman^25^ (see also ref. 26) according to which low SFs are continuously extracted to activate coarse representations of new objects in the visual field.

Moreover, we have discovered that ASD observers do not extract SFs across time like neurotypical observers: in the first 80 ms, high SFs around 25 cpi lead ASD observers to a higher accuracy than neurotypical observers, and these SFs are sampled differently across time in the two subject groups. An increased early sampling of high SFs in ASD subjects might explain the enhanced neural activity they exhibit early in visual processing in response to such SFs^8^. Furthermore, their initial simultaneous sampling of low and high SFs is consistent with the observation of a reduced segregation between neural responses to intermediate and high SFs in their early processing^9^,^10^.

Some authors have suggested that autistic perception could be explained by a deficit in the processing of global information^5^; others suggested that it could be explained better by a superiority in accessing local information^6^. Published findings on the subject are contradictory^1^,^7^,^27^. Importantly, our results do not show any difference in the extraction of low SFs (analogous to global information), and only a more immediate extraction of high SFs (analogous to local information). These results suggest that the difference in SF perception between neurotypicals and ASD observers relies specifically in the time course of extraction of high SFs. The local bias present in ASD perception might thus be the result of a more immediate access to fine visual information without the mandatory precedence of global information observed in neurotypicals.

The absence of a coarse-to-fine sampling in individuals with ASD might also be linked to abnormalities in top-down processing^20^,^22^. Prominent models of object recognition state that the extraction of low SFs activates an object representation that modulates the subsequent extraction of higher SFs in a top-down fashion^17^,^18^,^19^. In ASD observers, early extraction of low SFs cannot guide the sampling of the diagnostic higher SFs since high and low SFs are sampled equally early, soon after stimulus onset.

## Methods

### Participants

Forty-three neurotypical adults and twenty-one adults with ASD were recruited. Power analyses were not performed before the recruitment to determine the optimal sample sizes because of the use of a new experimental technique and unknown effect sizes. A sample size of approximately 20 was chosen for the ASD group because it is fairly common in studies of this clinical population; a larger sample size of approximately 40 was chosen for the neurotypical group to increase our statistical power. ASD participants were diagnosed by an expert psychiatrist or licensed clinical psychologist and the diagnosis had to be recently confirmed, with each having met the criteria for ASD within the past 3 years on the basis of the DSM-IV-TR^28^. In addition, most participants were also tested using either the AQ, ASAS-R or ADI-R questionnaire. Neurotypical participants were recruited on the campus of the Université de Montréal as a comparison group. Brief interviews ensured that none of the participants suffered from any mental or neurological disorder (other than ASD for the ASD group) and that they were free of medication.

Three neurotypical participants were excluded prior to the analysis: one because he did not complete the first block, one because the quantity of information he required to reach target performance was more than 3 standard deviations over the group mean, and one because his mean response time was more than 3 standard deviations over the group mean. One ASD participant was excluded because the quantity of information he required to reach target performance was more than 3 standard deviations over the group mean. The final ASD group thus included 20 participants (14 males; mean age = 27.10, SD = 8.72), and the final neurotypical group included 40 participants (19 males; mean age = 24.43, SD = 7.55). Subject groups did not differ significantly in age (*t*(58) = 1.17, *p* > 0.25) or gender (*p* = 0.17, Fisher’s exact test). Participants of both groups had or were completing a post-secondary diploma at the time of the study, and had normal or corrected-to-normal vision. The experimental protocol was approved by the ethics board of the University of Montreal’s Faculty of Arts and Sciences and the study was carried in accordance with the approved guidelines. Written informed consent from all participants was obtained after the procedure had been fully explained, and a monetary compensation was provided upon completion of the experiment.

### Materials

The experimental program ran on Mac Pro (Apple Inc.) computers in the Matlab (Mathworks Inc.) environment, using functions from the Psychophysics Toolbox^29^,^30^. All stimuli were presented on Asus VG278H monitors (1920 × 1080 pixels at 120 Hz), calibrated to allow linear manipulation of luminance. Luminance values ranged from 1.6 cd/m^2^ to 159 cd/m^2^. Chin rests were used to maintain viewing distance at 76 cm.

### Stimuli

Eighty-six grayscale object images were selected from the database used in Shenhav, Barrett, & Bar^31^ and from Internet searches. Images were 256 × 256 pixels (6 × 6 degrees of visual angle) and median object width was 220 pixels (SD = 47 pixels). The objects were cropped manually and pasted on a uniform mid-gray background. The SF spectrum of every object image was set to the average SF spectrum across all object images using the SHINE toolbox^32^. This preserved the most important spectral properties of natural objects while eliminating sources of undesired variance in the stimuli. Resulting images had a root mean square (RMS) contrast of about 0.20.

**Figure 2:**
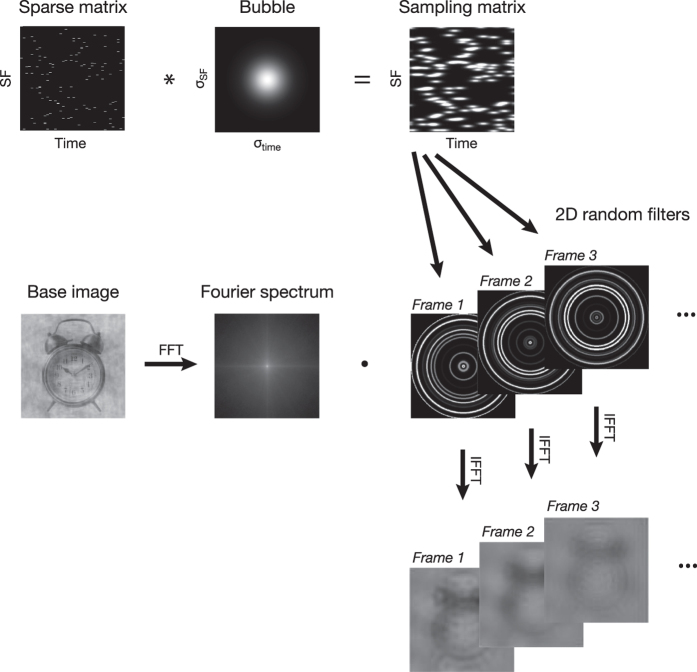
Illustration of the sampling method. On each trial, we randomly generated a matrix of dimensions 256 × 40 (representing respectively SFs and frames) in which most elements were zeros and a few were ones. We then convolved this sparse matrix with a 2D Gaussian kernel (a “bubble”). This resulted in the trial’s sampling matrix, shown here as a plane with a number of randomly located bubbles. Every column of this sampling matrix was then rotated around its origin to create isotropic 2D random filters. Finally, these 2D random filters were dot-multiplied by the base image’s spectrum and inverse fast Fourier transformed to create a filtered version of the image for every video frame.

On each trial, participants were shown a short video (333 ms) consisting of an object image with random SFs gradually revealed at random time points (e.g., Video S1; Video S2). To create these stimuli, we first randomly generated, on each trial, a matrix of dimensions 256 × 40 (representing respectively SFs from 0.5 to 128 cycles per image or cpi, and frames, each lasting 8.33 ms) in which most elements were zeros and a few were ones. The number of ones was adjusted on a trial-by-trial basis to maintain performance at 75% correct. We then convolved this sparse matrix with a 2D Gaussian kernel (a “bubble”; σ_SF_ = 1.5 cpi; σ_time_ = 15 ms). This resulted in the trial’s sampling matrix: a SF × time plane with randomly located bubbles. Every column of this sampling matrix was then rotated around its origin to create isotropic 2D random filters. Finally, these 2D random filters were dot-multiplied by the base image’s spectrum and inverse fast Fourier transformed to create a filtered version of the image for every video frame (see Fig. 2 for an illustration of this method). Thus, a SF × time bubble centered on the SF of 10 cpi and the frame 6 in the sampling matrix would reveal this SF on this frame (and, to a lesser extent, the neighboring SFs on the neighboring frames) *in the entire image*. To ensure accurate luminance display, we applied noisy-bit dithering to the final stimuli^33^.

This random sampling method was preferred to other methods with lower dimensionality because it allowed us to perform a systematic search of the SF × time space and thus to precisely infer which SFs in which time frames led to an accurate response. Moreover, this method is unbiased, as it does not require the selection of a small number of arbitrary cut-offs.

### Procedure

After they had completed a short questionnaire for general information (age, sex, lateralisation, education, language), participants sat in front of a computer monitor, in a dim-lighted room. Participants completed two 500-trial blocks on the first day and two more on another day. Each trial comprised the following consecutive events, on a mid-gray background: a fixation cross (300 ms), a uniform mid-gray field (200 ms), the video stimulus (333 ms), a fixation cross (300 ms), a uniform mid-gray field (200 ms), and an object name that remained on the screen until a response was provided by the participant or for a maximum of 1 s. Subjects were asked to indicate whether the name matched the object (it did 50% of the time) with a keyboard key press as rapidly and accurately as possible. The number of bubbles (i.e. the quantity of information revealed during the whole video) was adjusted on a trial-by-trial basis using a gradient descent algorithm to maintain performance at 75% correct.

### Regression analysis

Before performing the regressions, accuracies and response times were transformed in z-scores for each object (to minimize variability due to psycholinguistic factors), for each 500-trial block (to minimize variability due to task learning), and for each subject (to minimize residual individual differences in performance). Trials associated with z-scores over 3 or below -3 (either in accuracy or response time) were discarded (2.13% of trials overall). Sampling matrices were also transformed in z-scores on each trial; this equalized the importance given to all trials, irrespective of the number of bubbles used on these trials.

Following the regressions, the subject-wise maps of regression coefficients were convolved with a Gaussian kernel (σ_SF_ = 3.5 cpi; σ_time_ = 42 ms) and transformed in z-scores with a bootstrapped sample, before computation of the *t* statistics. The two-sample *t* statistics were computed according to Welch’s formula, which does not assume equal variances of the populations. Statistical significance of the *t* maps was assessed with a Pixel test^34^, which controls the FWER while taking into account the correlation in the data. All reported statistical test results are two-tailed.

## Additional Information

**How to cite this article**: Caplette, L. *et al*. Atypical Time Course of Object Recognition in Autism Spectrum Disorder. *Sci. Rep.* **6**, 35494; doi: 10.1038/srep35494 (2016).

## Supplementary Material

Supplementary Information

Supplementary Video S1

Supplementary Video S2
