# Supplementary material for: Atypical Time Course of Object Recognition in Autism Spectrum Disorder
Source: Sci Rep. 2016 Oct 18;6:35494. doi: 10.1038/srep35494 (PMC5067503; doi:10.1038/srep35494)
Supplement: Supplementary Information [file srep35494-s1.pdf]

Atypical Time Course of Object Recognition in Autism Spectrum Disorder

(Supplementary Information File)

Laurent Caplette[1*], Bruno Wicker[2], & Frédéric Gosselin[1]

[1]Département de psychologie, Université de Montréal

[2]Institut de Neurosciences de la Timone, CNRS UMR 7289, Aix-Marseille Université

*Corresponding author. Email: laurent.caplette@umontreal.ca

Supplementary Video Legends

Video S1. Example of a stimulus.

Video S2. Example of a stimulus (slowed down 10x).
